# HIF-1α expression is associated to cell proliferation and ECM production in temporomandibular joint osteoarthritis

**DOI:** 10.1371/journal.pone.0361577

**Published:** 2026-11-10

**Authors:** Li Liu, Xiaoying Shi, Xingxing Li, Yunhong Lin, Hefeng Yang, Jun Zhang, Chun Yang

**Affiliations:** 1 Department of Prosthodontics, Kunming Medical University School and Hospital of Stomatology, Kunming, Yunnan, China; 2 Yunnan Key Laboratory of Stomatology, Kunming, Yunnan, China; 3 Department of Pediatric Dentistry, Kunming Medical University School and Hospital of Stomatology, Kunming, Yunnan, China; 4 Department of Basic Science of Stomatology, Kunming Medical University School and Hospital of Stomatology, Kunming, Yunnan, China; State Key Laboratory of Oral & Maxillofacial Reconstruction and Regeneration, Key Laboratory of Oral Biomedicine Ministry of Education, Hubei Key Laboratory of Stomatology, School and Hospital of Stomatology, Wuhan University, CHINA

## Abstract

Temporomandibular joint osteoarthritis (TMJOA) is a complex joint disorder characterized by progressive degradation of the cartilage matrix and apoptosis of chondrocytes; however, hypoxia-inducible factor 1α (HIF-1α) serves a positive role in maintaining the stability of the articular cartilage matrix. Therefore, the present study aimed to determine the expression levels of HIF-1α in the cartilage tissue of rats with TMJOA, and to explore its association with the proliferation of chondrocytes and the formation of cartilage matrix. Experimental animals were subjected to occlusal interference treatment for periods ranging from 2 to 10 weeks to induce osteoarthritis (OA)-like pathological changes. Subsequently, various histochemical methods were employed to systematically evaluate the degree of articular cartilage damage in the TMJOA rat model. The expression levels of HIF-1α, proliferating cell nuclear antigen (PCNA), collagen type II (COLII) and aggrecan in cartilage tissues of rats with OA were analyzed using immunohistochemistry and immunofluorescence techniques. Western blotting and immunofluorescence were used to quantify the expression levels of HIF-1α, PCNA, Aggrecan and COLII in primary chondrocytes under hypoxic conditions (1% O2). EdU was used to assess the chondrocyte proliferation. From week 4 onward, a reduction in HIF-1α expression levels were detected in the lesioned cartilage of the TMJOA rats. Furthermore, the percentage of HIF-1α-positive chondrocytes was found to be significantly negatively associated with the OA score. This study demonstrated that the expression pattern of HIF-1α was consistent with cell proliferation and extracellular matrix production, and was negatively correlated with the severity of condylar cartilage degeneration.

## Introduction

The temporomandibular joint (TMJ) is a complex synovial joint that enables both hinge-like and gliding movements of the mandible relative to the cranial fossa [1]. The condyle of the TMJ is lined with fibrocartilage, a dense, avascular connective tissue. This tissue has limited regenerative potential [2]. During TMJ movement, the fibrocartilage provides functional cushioning between the condyle and the opposing bony surface of the glenoid fossa [3]. However, when TMJ osteoarthritis (TMJOA) affects the cartilage of the TMJ, the limited regenerative capacity of fibrocartilage poses a challenge for clinical treatment [4,5]. Owing to the unclear molecular mechanisms underlying cartilage degradation and extracellular matrix (ECM) loss in TMJ-OA, prevention or management of joint damage induced by TMJOA remains a notable clinical challenge [6]. Research has indicated that dysregulation of hypoxic homeostasis can lead to cartilage dysfunction and subchondral bone alterations, which ultimately impair chondrocyte viability and promote the development and progression of osteoarthritis (OA) [7].

Articular cartilage maintains a physiological hypoxic microenvironment and corresponding metabolic state due to its avascular nature [8]. The oxygen level balance in the cartilage microenvironment is crucial for maintaining tissue integrity and preventing the progression of OA [9–13]. A key downstream effector of hypoxia is the hypoxia-inducible factor 1α (HIF-1α) [14], which is generally considered a master regulator of chondrocyte adaptation to hypoxia, promoting cell survival, matrix synthesis and glycolytic metabolism [15]. HIF-1α was detected in the nuclear extracts of chondrocytes isolated and cultured from both normal cartilage and OA cartilage [16,17]. HIF-1α stabilization has been shown to induce metabolic reprogramming that fortifies the collagen matrix, thereby potentially mitigating degradation [18].

Chondrocytes are the only resident cell type in cartilage, maintaining the structural and functional integrity of cartilage tissue by regulating the metabolism of the ECM [19]. It has been demonstrated that a decline in chondrocyte function or number leads to cartilage degeneration. Furthermore, chondrocytes have a relatively slow proliferation rate [20], and chondrocyte death is a recognized pathological hallmark of OA [21]. Articular chondrocytes grow in a hypoxic environment, and HIF-1α serves a key role in their survival [22].

The proliferation of chondrocytes and the production of ECM are essential for maintaining the integrity of cartilage. To date, the importance of HIF-1α in cartilage assessment has gained increasing recognition due to its essential role in maintaining cartilage homeostasis; however, its downstream signaling mechanisms in TMJOA remain unclear [23,24]. The present study aimed to explore the expression pattern of HIF-1α in occlusal interference-induced TMJOA rat model at different time points, its association with cell proliferation and ECM generation.

## Materials and methods

### Establishment of TMJOA model in rats

The present study used sixty 8-week-old male Sprague-Dawley (SD) rats weighing 160–180g from the experimental animal center of Kunming Medical University. They were housed in a specific pathogen-free environment and were kept in separate cages under a 12-h light/dark cycle, with the cage temperature maintained at 20–22 ˚C and humidity at approximately 50%. The rats were fed a standard diet and provided with sterilized drinking water. After one week of acclimatization feeding, they were included in the experiment. All experiments were carried out in compliance with the ARRIVE guidelines and with the approval of the Ethical Committees of Kunming Medical University (approval no. kmmu20240001). Rats were then randomly assigned to a control group (n = 10) and an occlusal disturbance (OD) group (n = 50). In the OD group, anesthesia was induced with 5% isoflurane and maintained with 1–3% isoflurane in 100% oxygen, an anterior dental elevation was applied to induce malocclusion for establishing a TMJOA model, as previously described [20]. The control group rats received no special treatment. OD rats with occlusal disorder were deeply anesthetized with isoflurane (5% induction, 2–4% for maintenance) subsequently euthanized by cervical dislocation, performed by trained and experienced personnel at 2 weeks (n = 10), 4 weeks (n = 10), 6 weeks (n = 10), 8 weeks (n = 10), and 10 weeks (n = 10), respectively.

The harvested TMJs were dissected and fixed overnight in 4% paraformaldehyde. After decalcification in 10% ethylenediaminetetraacetic acid (EDTA) solution (pH 7.2–7.4), the samples were embedded in paraffin, cut in the sagittal plan in serial sections at 5μm thickness for subsequent experiments.

### Histological staining

After dewaxing and gradient hydration, standard hematoxylin and eosin (H&E), safranin O and fast green (Beyotime Institute of Biotechnology) and alcian blue staining (C0155S, Beyotime Institute of Biotechnology) was used to assess the health status of cartilage and evaluate the effectiveness of cartilage repair. For TMJOA scoring, Safranin O/Fast Green-stained sections (n = 5–6/group, based on previous studies [25,26] were evaluated using an adaptation to the Modified Mankin Score by three blinded graders. The Modified TMJ Mankin Score was adapted to include additional metrics of subchondral bone. To assess the extent of joint injury histologically, the OARSI scale was adopted [27,28]. Two qualitative trained observers (unknown sample sources) were identified according to H&E staining analysis of cartilage fragments.

### Immunohistochemistry and immunofluorescence staining

The tissue sections were sequentially dewaxed and rehydrated. After being incubated with 3% hydrogen peroxide (Beyotime Institute of Biotechnology) for 15 min, the slides were blocked with 5% BSA (Beyotime Institute of Biotechnology) at room temperature for 2 h and incubated with primary antibodies against HIF-1α (1:500, EPR16897, Abcam), proliferating cell nuclear antigen (PCNA) (1:3000, 10205–2-AP, Proteintech), Aggrecan (1:200, 13880–1-AP, Proteintech) and COLII (1: 500, PA5–99159, Invitrogen) in blocking buffer at 4 ˚C overnight. In the negative control groups, the primary antibody was replaced by non-immune serum. Then react with goat anti-rabbit HRP secondary antibody (KIT-5010, Maixin) at room temperature for 15 min. The tissue sections were incubated in 3,3’-diaminobenzidine (DAB-0031, Maixin) for 50 seconds to develop the color of the bound immunocomplexes. Hematoxylin was used for counterstaining using light microscopy.

For immunofluorescence, samples were incubated overnight at 4 ˚C with primary antibodies against HIF-1α (1:500, EPR16897, Abcam), PCNA (1:200, 10205–2-AP, Proteintech), Aggrecan (1:200, 13880–1-AP, Proteintech) and COLII (1: 200, 28459–1-AP, Proteintech), then with goat anti-Rabbit IgG (H + L) Cross-Adsorbed Secondary Antibody, Alexa Fluor 488/555 (1:200, A-11008/ A-21428, Invitrogen) for 1 h at room temperature in the dark. Following DAPI staining of the nuclei, images were captured aconfocal laser scanning microscope (Leica Biosystems).

### Cell culture and identification

Primary mandibular condylar chondrocytes (MCCs) were isolated from the TMJ of healthy 1–3day old SD rats (n = 10 pups from 3 litters). Only male pups were used. After euthanasia, mandibular bones were dissected, and condylar cartilage was separated from the bone using surgical blades. The cartilage tissues were rinsed with phosphate-buffered saline (PBS) and sequentially digested with 0.25% trypsin for 30 min, followed by 2 mg/mL type II collagenase solution for 2 h to remove residual extracellular matrix. Single-cell suspensions were cultured in Dulbecco’s Modified Eagles Medium (DMEM) (Hyclone) containing 20% fetal bovine serum (Gibco) and 1% penicillin-streptomycin (P/S) (Hyclone). To preserve chondrocyte phenotype, only passage 1–2 cells were utilized for subsequent experiments.

The chondrocytes were seeded at a cell density of 2 × 10^5^ cells per well and fixed in 4% paraformaldehyde for 30 min at room temperature, and stained with Alcian Blue, toluidine blue and Safranin O. Culture medium in hypoxic conditions was replaced every 2 days.

### Cellular immunofluorescent staining

After culture and treatment, chondrocytes were fixed with 4% paraformaldehyde for 30 min at room temperature, permeabilized with 0.1% Triton X-100, and then blocked with 10% goat serum (Beyotime Institute of Biotechnology) for 60 min at room temperature. The cells were incubated overnight at 4 ˚C with primary antibodies against HIF‑1α (1:500, EPR16897, Abcam), Aggrecan (1:200, 13880–1-AP, Proteintech), and COLII (1: 500, PA5–99159, Invitrogen). Subsequently, the cells were incubated using secondary antibody conjugated with fluorescence (Beyotime Institute of Biotechnology) for 1 h at room temperature in the dark, followed by nuclear staining with DAPI (Beyotime Institute of Biotechnology) for 5 min. Finally, the cells were observed and images captured using a fluorescence microscope (Leica Biosystems).

### Western blotting

Treated chondrocytes were collected and washed three times with PBS. The cells were then lysed using RIPA buffer containing 1mM PMSF. Equal protein loading was ensured quantification using Bradford Protein Assay Kit (Beyotime Institute of Biotechnology) a prior to gel electrophoresis. About 30 μg of protein was dropped in the single hole of the 10% or 12% SDS‑polyacrylamide gels and electrophoresed to isolate target proteins. Next, the proteins were transferred to PVDF membranes under the condition of 300mA and 90 min. After blocking with 5% skim milk/TBST for 45 min at room temperature, membranes were incubated overnight at 4 ˚C with primary antibodies: anti-HIF‑1α (1:1000, EPR16897, Abcam), anti-COLII (1: 1000, PA5−99159, Invitrogen), anti-Aggrecan (1:1000, 13880–1-AP, Proteintech), anti-PCNA (1:20000, 10205–2-AP, Proteintech), and anti- β‑tubulin (1:6000, 10094–1-AP, Proteintech). The membranes were then washed with TBST and incubated with corresponding secondary antibodies (1:5000, RGAR001, Proteintech) at room temperature for 90 min. Finally, immunoreactive protein bands were detected using a chemiluminescent ECL system (Biosharp Life Sciences).

*5-ethynyl-2-deoxyuridine (EdU) staining.* The Cell-Light EdU Apollo 567 *in vitro* kit (Ribo Biotechnology Co., Ltd.) was used to assess the proliferation of chondrocytes in accordance with the manufacturer’s instructions. Specifically, chondrocytes (2x10^4^ per well) were cultured in a 12-well glass culture plate and then incubated with 50μmol/l EdU (1:1000) for 12 h at room temperature. Subsequently, the cells were fixed with 4% formaldehyde for 20 min at room temperature, and permeabilized with 0.5% Triton X-100 at room temperature for 15 min. Then, 100 μl of the Apollo® reaction mixture was added to each well, and the cells were incubated in the dark for 30 min at 37 ˚C. After three washes with PBS, the cells were stained with DAPI for 20 min at room temperature to visualize the cell nuclei, and the EdU-labeled cells were observed using a laser scanning confocal microscope (Leica Biosystems) and normalized against the total number of DAPI-stained cells.

### Statistical analysis

Data were analyzed using GraphPad Prism (version 9, Dotmatics) and expressed as mean ± standard deviation from three independent biological replicates. Differences between the two groups were analyzed using the unpaired Student’s t-test. Comparisons between multiple groups was performed using one-way ANOVA followed by Dunnett's multiple comparisons test. *P < 0.05* was considered to indicate a statistically significant difference.

## Results

### TMJOA-like lesions after occlusal induction in rat TMJs

To mimic human TMJOA conditions, a rat OA model was used. H&E staining showed that condylar cartilage in the experimental group showed structural and degenerative changes compared with the control. At 4, 6, 8 and 10 weeks, the cartilage in the OD group was thinner when compared with that of the control group (Fig 1A). The superficial cartilage showed disorder (Fig 1A). Staining with safranin O revealed that in the control group, the distribution of proteoglycans was uniform and abundant, and a clear boundary between the fibrous layer and the cartilaginous layer. However, in the OD group at 4 weeks, cartilage degeneration had occurred, accompanied by extensive loss of proteoglycan regions and a decrease in the total number of chondrocytes (Fig 1A). Alcian blue staining showed the staining was lighter at 4 weeks in the OD group compared with that in the control group. Masson staining revealed that the amount of unmineralized bone was increased, in OD group at 4 weeks compared with the control groups (Fig 1C). In the OD group, the Mankin score (Fig 1B) and OARSI score (Fig 1D) increased from the 4th week in the OD group. These histological staining results demonstrated that progressive degenerative changes in the condylar cartilage became more pronounced with the extended time course of the animal model. In the OD group at 2 weeks, no significant damage was observed in the condylar cartilage.

**Fig 1:**
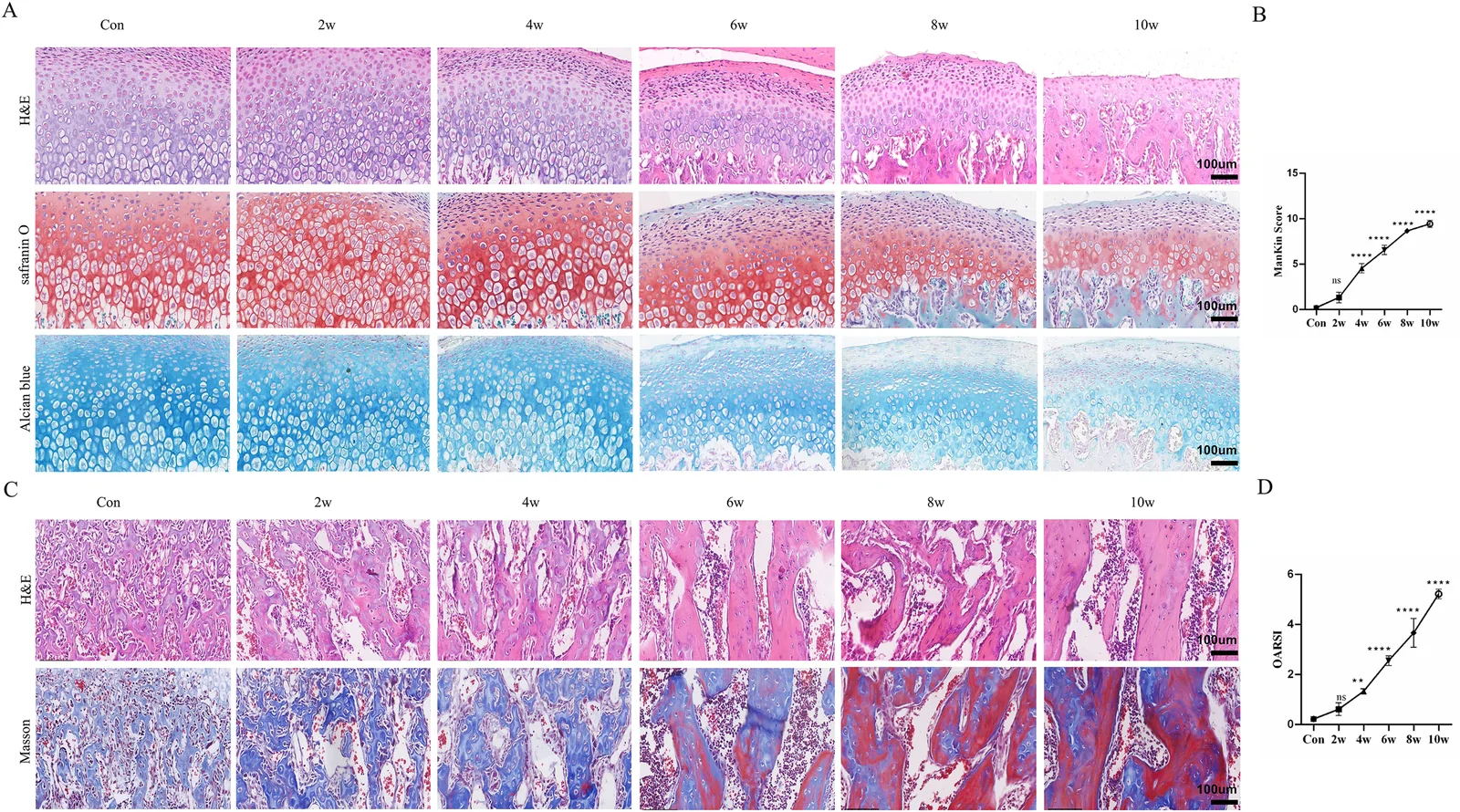
OA-like lesions in the condyle of TMJOA animal model. (A) H&E staining, safranin O-fast green staining and alcian blue staining. (B) Mankin score for evaluating the degree of condylar cartilage damage after modeling. **P < 0.01, ***P < 0.005, ****P < 0.0001.(C) H&E staining and Masson staining. (D) OARSI score statistics. **P < 0.01, ***P < 0.005, ****P < 0.0001. OA, osteoarthritis; TMJOA, temporomandibular joint OA; OARSI, OA research society international.

### Expression levels of HIF1α and chondrocyte proliferation in OD rat model

The degeneration of articular cartilage directly affects the chondrocyte microenvironment, particularly the adaptability of cells to hypoxic conditions. The expression of HIF‑1α is closely associated with low‑oxygen environments; therefore, this factor potentially serves an important role in maintaining chondrocyte survival and basic homeostasis under the physiologically hypoxic conditions of normal articular cartilage [29]. Immunohistochemical staining and immunofluorescence detection of HIF‑1α was performed on condylar cartilage sections. As shown in Fig 2A-D, HIF‑1α expression increased at 2 weeks and gradually decreased after 4 weeks, a trend that aligned with chondrocyte proliferation. These findings suggested that HIF‑1α may exert a positive role by promoting cell proliferation in early TMJOA, and its decreased expression could be an important stimulus driving TMJ degeneration.

**Fig 2:**
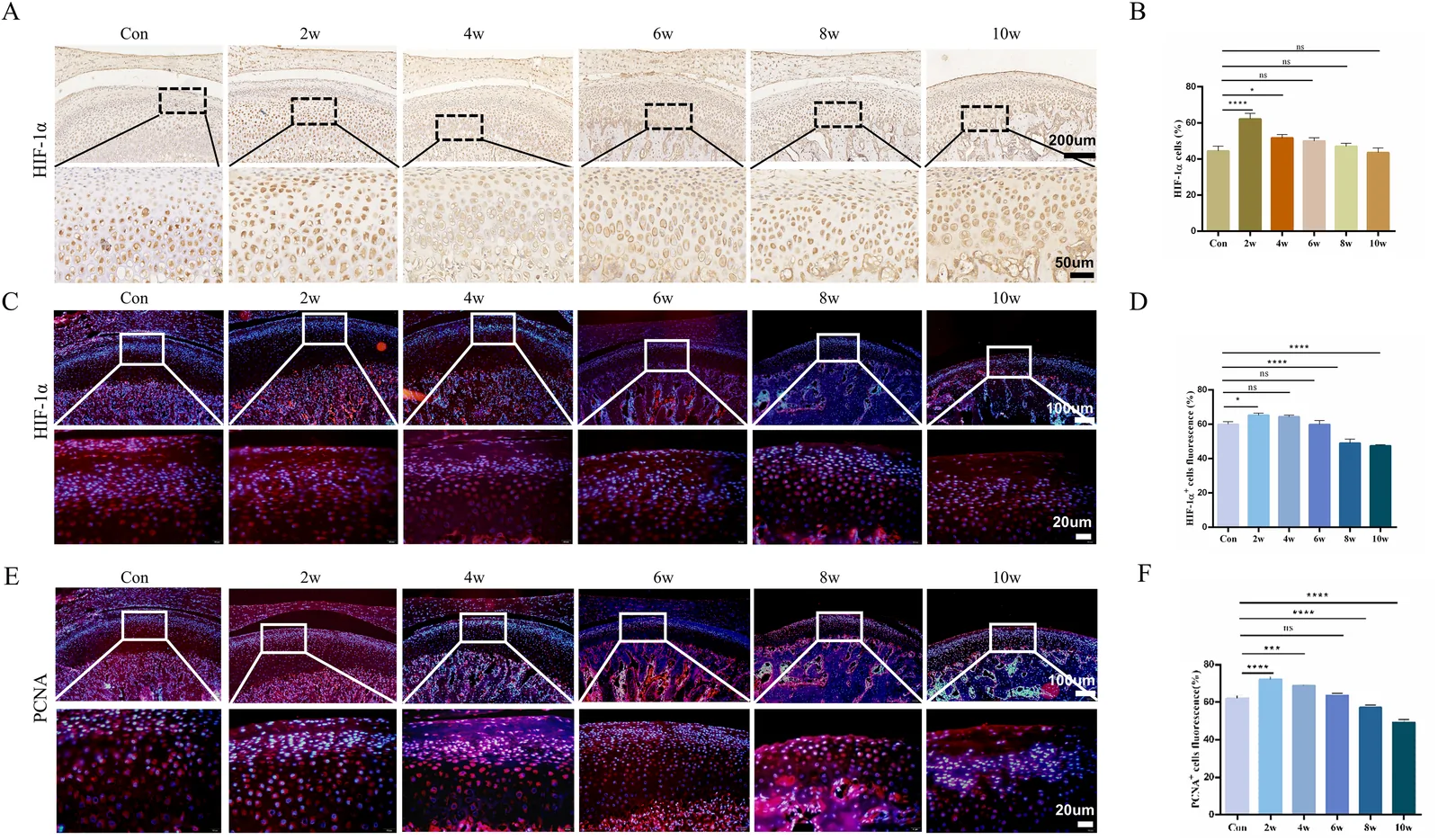
Expression levels of HIF-1α and PCNA in the condyle of TMJOA animal model. (A) Immunohistochemical staining was used to analyze the expression of HIF-1α in rat condylar cartilage. (B) Positive immunohistochemical analysis of HIF-1α, *P < 0.05, **P < 0.01, ***P < 0.001, ****P < 0.0001. (C) Immunofluorescence staining was used to analyze the expression of HIF-1α in rat condylar cartilage. (D) Immunofluorescence positive rate analysis of HIF-1α. *P < 0.05, ****P < 0.0001. n = 3, Scale bar, 100μm. (E) Immunofluorescence staining was used to analyze the expression of PCNA in rat condylar cartilage. (F) Immunofluorescence positive rate analysis of PCNA, ***P < 0.001, ****P < 0.0001. n = 3, Scale bar, 100μm. ns, not statistically significant; TMJOA, temporomandibular joint osteoarthritis; HIF-1α, hypoxia-inducible factor 1α; PCNA, proliferating cell nuclear antigen.

### Expression levels of condylar cartilage matrix protein in the OD rat model

The expression of cartilage matrix proteins in the TMJ was evaluated using immunohistochemistry and fluorescence assays (Fig 3). At the 2-week time point, the expression of Aggrecan and COLII in the condylar cartilage were increased in the occlusal interference group compared with the control group. From week 4, the expression levels of Aggrecan and COLII in the OD group was lower when compared with that of the control group. These results indicated that open occlusal interference after 4 weeks led to reduced expression of Aggrecan and COLII in the cartilage layer, similar to that of HIF-1α expression. Therefore, HIF-1α expression was associated with the expression of synthetic proteins in TMJ cartilage.

**Fig 3:**
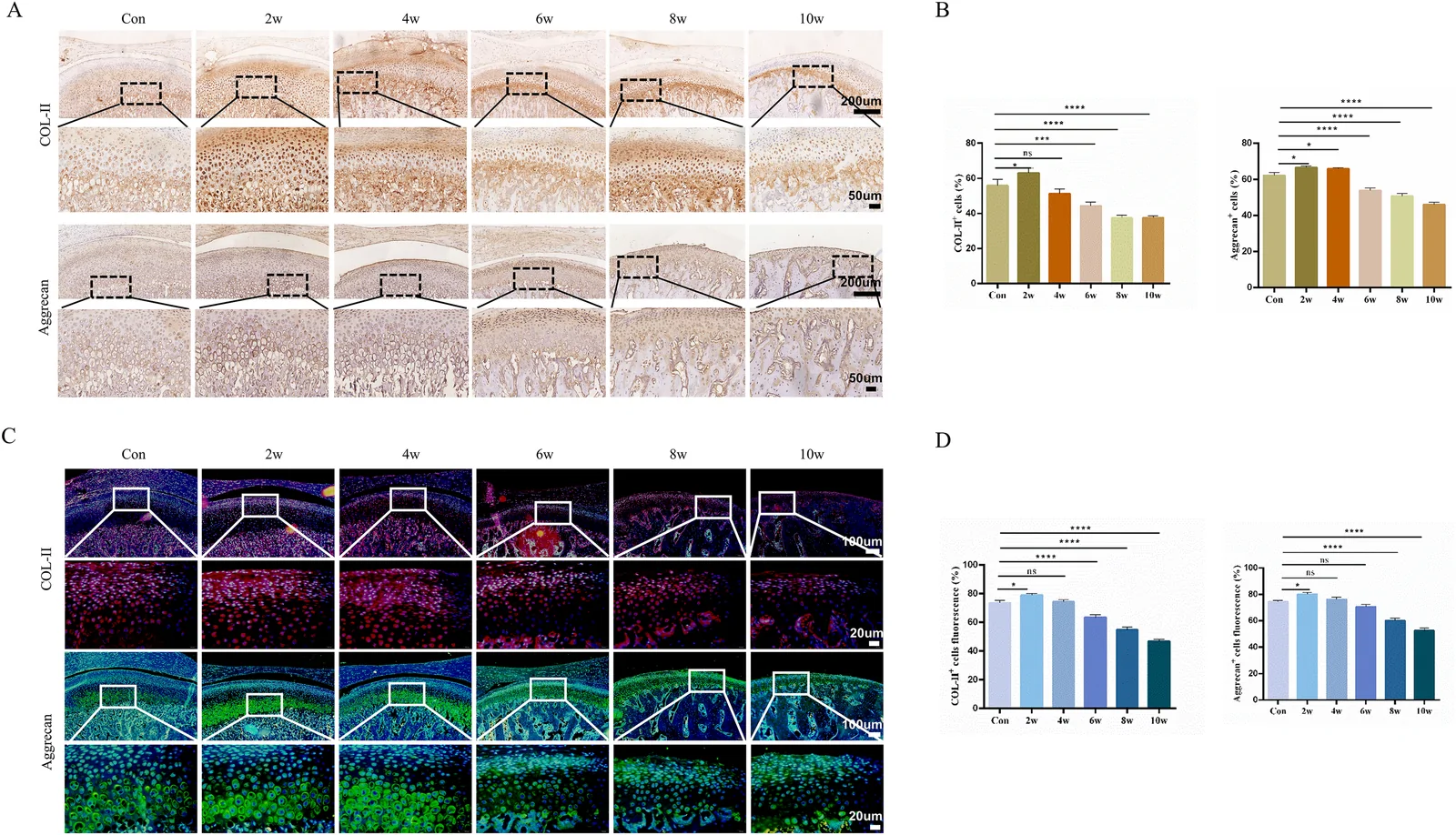
Expression levels of Aggrecan and COLII in the condyle of TMJOA animal model. (A) Immunohistochemical staining of COLII and Aggrecan in rat condylar cartilage. (B) Immunohistochemical positive analysis of COII and Aggrecan, *P < 0.05, ***P < 0.001, ****P < 0.0001. n = 3, Scale bar, 200μm. (C) Immunofluorescence staining of COLII and Aggrecan in rat condylar cartilage. (D) Immunofluorescence positive analysis of COLII and Aggrecan, *P < 0.05, ***P < 0.001. n = 3, Scale bar, 200μm. ns, not statistically significant; TMJOA, temporomandibular joint osteoarthritis; COLII, type II collagen.

### HIF-1α expression levels and cell proliferation in MCCs under hypoxia

HIF-1α proteins only exist under hypoxic conditions [30], therefore, to mimic hypoxic environment, MCCs were incubated in Billups-Rothenberg (1%O_2_ and 0.5%O_2_) over a 0–24 h period (Fig 4B). To characterize MCCs under hypoxia, we observed time-dependent changes in the morphology and proliferation rate of MCCs. MCCs from the rat condyle are palisade-like in appearance and grow rapidly [31]. Immunofluorescence showed positive expression of COLII and SOX9, which were analyzed by toluidine blue, Alcian Blue and Safranin O staining (Fig 4A) [32–34]. EdU assays revealed that cell proliferation rates peaked at 6 h followed by a gradual decline after 12 h under hypoxic condition (Fig 4C-D). The expression of PCNA was consistent with EdU (Fig 4E).

**Fig 4:**
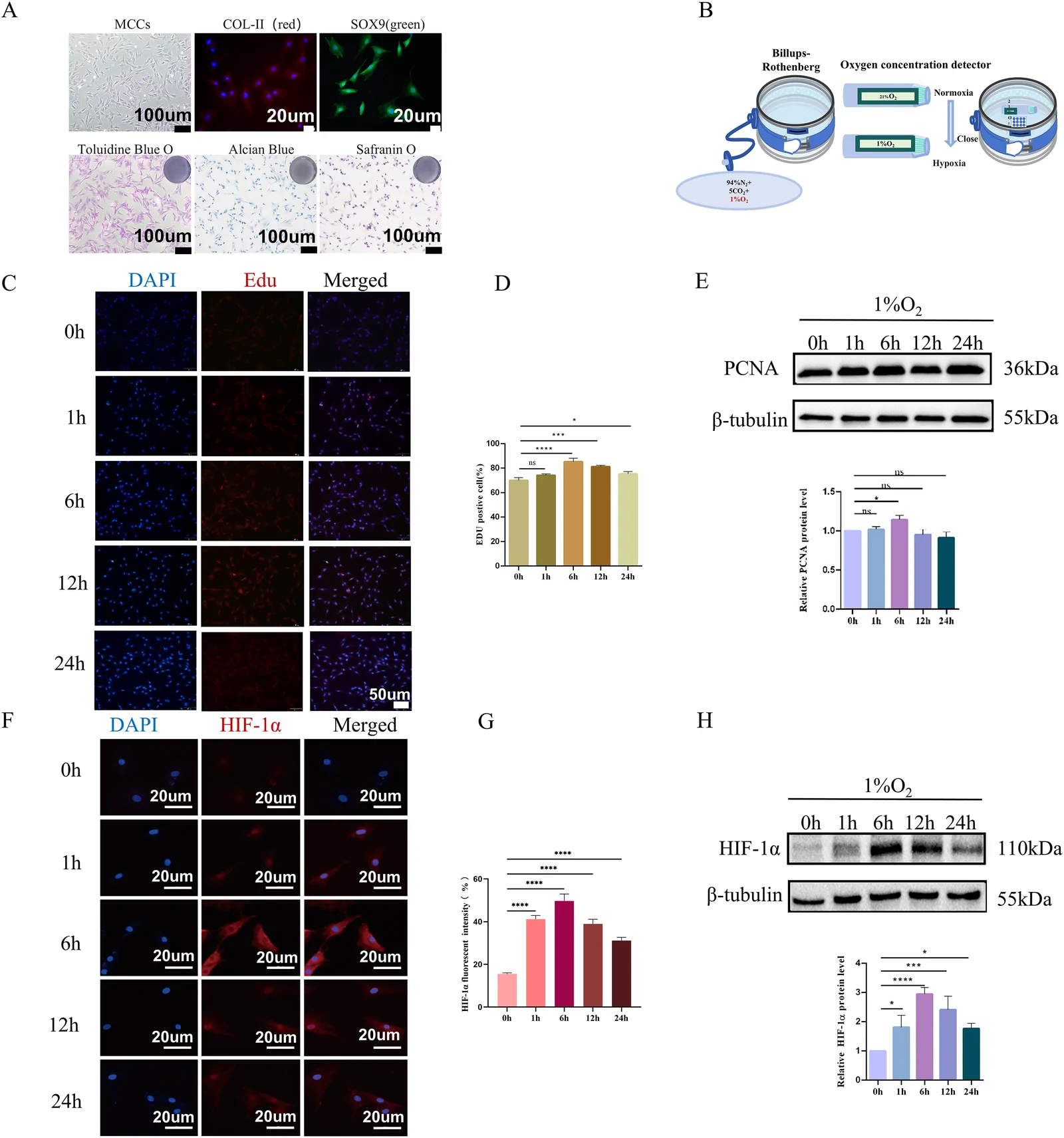
Expression levels of HIF-1α and proliferation after hypoxic culture of MCCs. (A) Morphology of MCCs under light microscopy, immunofluorescence of COLII, SOX9, and Toluidine blue, alcian blue and Safranin O staining of MCCs. Scale bar, 200 μm; Scale bar, 100 μm. (B) Schematic diagram of hypoxia device. (C) EdU was used to detect the changes in the proliferation rate of MCCs under hypoxic conditions. (D) Quantitative statistics of EdU levels in hypoxia at different time-points, *P < 0.05, ***P < 0.001, ****P < 0.0001. n = 3. (E) The protein and semi-quantitative statistical chart of PCNA in hypoxia at different time-points, *P < 0.05. n = 3. (F) Immunofluorescence of HIF-1α in hypoxia; Scale bar, 50μm. (G) Immunofluorescence positive analysis of HIF-1α, ****P < 0.0001. n = 3. (H) The protein and semi-quantitative statistical diagram of HIF-1α in hypoxic chamber. *P < 0.05, ***P < 0.001, ****P < 0.0001. n = 3. ns, not statistically significant; TMJOA, temporomandibular joint osteoarthritis; HIF-1α, hypoxia-inducible factor 1α; PCNA, proliferating cell nuclear antigen; MCCs, mandibular condylar chondrocytes.

HIFs serve as central regulators that activate hypoxia-responsive genes and help restore cellular oxygen homeostasis [35]. The HIF-1α protein level exhibited a biphasic pattern, characterized by an initial significant upregulation, peaking at 6 hours, followed by a gradual decline under 1% O_2_ conditions throughout the observation period. (Fig 4H). The result of fluorescence showed that HIF-1α protein levels peaked at 6 h, followed by a progressive decline throughout the observation period (Fig 4F-G). However, the expression trend of HIF-1α at 0.5% oxygen concentration was unstable (S1 Fig), so we chose 1% oxygen concentration for our subsequent experiments. The experimental results are consistent with those from the *in vivo* experiments.

### Cartilage matrix protein in MCCs under hypoxia

The protein levels of Aggrecan and COLII exhibited a biphasic response characterized by transient upregulation followed by gradual attenuation in hypoxic condition (Fig 5A-B). Immunofluorescence analysis indicated that COLII and Aggrecan expression was highest at 6 h, followed by a gradual decrease. These results confirmed that hypoxia affected the cell proliferation, synthesis of ECM in chondrocytes and that HIF-1α expression was associated to cell proliferation and ECM production in the early hypoxia.

**Fig 5:**
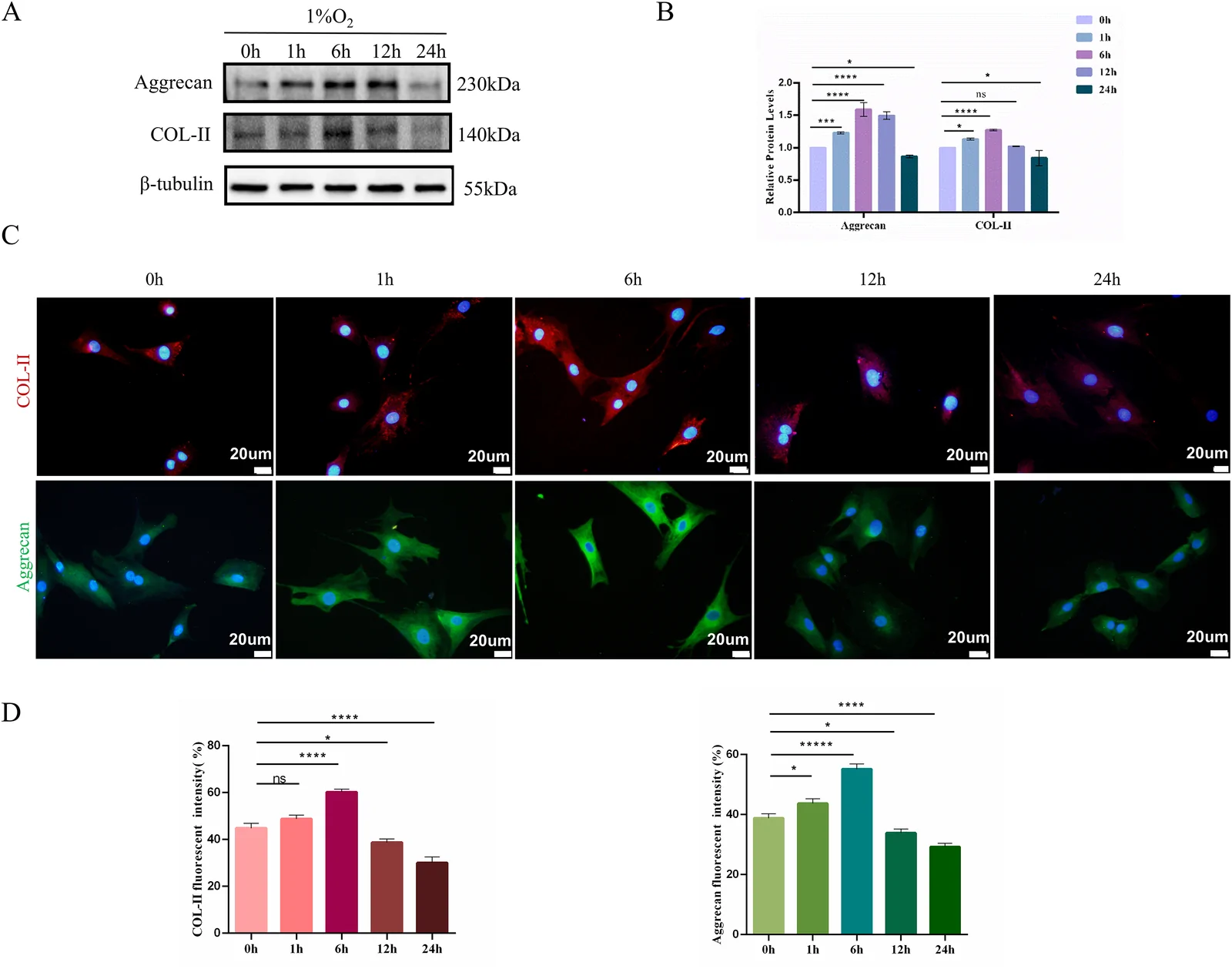
Expression levels of Aggrecan and COLII after hypoxic culture of MCCs. (A) The protein levels of Aggrecan and COLII under hypoxic conditions. (B) Quantitative statistics of Aggrecan and COLII, *P < 0.05, ***P < 0.001, ****P < 0.0001, n = 3.n = 3. (C) Immunofluorescence of Aggrecan and COLII in hypoxic condition. Scale bar, 20μm. (D) Immunofluorescence positive analysis of Aggrecan and COLII, ****P < 0.0001, n = 3. ns, not statistically significant, MCCs, mandibular condylar chondrocytes; COLII, type II.

## Discussion

TMJ-OA, a biomechanically-driven degenerative disorder, is pathologically characterized by progressive articular cartilage degradation, subchondral bone remodeling with sclerosis, chronic synovial inflammation, and osteophytic proliferation [36]. To investigate the pathogenesis of TMJ-OA, the present study established a rat model by anterior teeth overbite interference, a reproducible method that simulates biomechanical stress-induced joint disorders [37]. After 2 weeks of occlusal pressure, structural changes indicative of repair was observed, which developed into significant cartilage degradation from the 4th to the 10th week, as demonstrated by elevated Mankin and OARSI scores. In OA, the oxygen concentration in the joint fluid decreases. This leads to a reduction in the oxygen content within the cartilage of the joint, in which chondrocytes undergo various changes in their state, including alterations in proliferation, viability, and secretory patterns [38,39].

The condylar fibrocartilage is avascular, thereby maintaining chondrocytes in a physiological hypoxic niche [40]. Immunofluorescence analysis indicated that COLII and Aggrecan expression levels were highest at 2 weeks followed by a gradual decrease, and significant first increased and then decreased in HIF-1α protein levels in the OD group was observed, indicating an impaired hypoxic response. The changes in HIF-1α expression levels were negatively associated with the pathological alterations in the condylar cartilage. HIF-1α expression levels increased during cartilage repair and decreased when cartilage destruction worsened. To decouple the direct effects of hypoxia from complex *in vivo*, an *in vitro* system was also utilized in the present study. Although the precise oxygen tension in cartilage *in vivo* is uncertain, initial measurements and mathematical models estimate oxygen levels to be as low as 1%, with an average 5% [41]. Rat condylar chondrocytes were cultured in a hypoxic environment to simulate the microenvironment of the temporomandibular joint, and the changes in their phenotype over time were analyzed. The present study demonstrated that HIF-1α expression levels increase during the early stages of OA and under 1% O_2_, but decreases in later stages. This may be attributed to the fact that the protein expression of HIF-1α in cells rapidly increases within a short period after transitioning from normoxia to hypoxia, typically peaking within 4–8 h [42]. However, when hypoxia persists for an extended duration, prolyl hydroxylase domain-containing protein is activated through negative feedback, leading to the degradation of intracellular HIF-1α protein [43]. At the same time, the expression level of HIF-1α is consistent with the severity of OA and aligns with the trends of chondrocyte proliferation and ECM formation. This suggests that it may exert anabolic effects by promoting the expression of COLII and Aggrecan. The observed late-stage decrease in HIF-1α expression level during the present study is an intriguing finding, though its precise etiology remains unclear based on the current experimental design. It may potentially result from the degradation of intracellular HIF-1α protein due to negative feedback activation of prolyl hydroxylase domain-containing proteins during prolonged hypoxia [44]. The reduction in HIF-1α expression at the late stage might reflect actual cell death or a decrease in cell viability at later time points [45,46]. Alternatively, it could signify an exhaustion of adaptive responses, where initial protective mechanisms are no longer sustainable. We cannot rule out the involvement of oxidative stress, which could accumulate and impair cellular function over time [23]. Finally, a progressive failure of hypoxic signaling pathways, such as impaired HIF-1α stability or transcriptional activity, could also lead to this decrease [47]. Future time-course experiments incorporating specific inhibitors are warranted to investigate these possibilities.

Composed of chondrocytes and an ECM, articular cartilage relies on its singular cell type, the chondrocyte, to sustain its structure. This is achieved by the chondrocytes’ ability to orchestrate the homeostasis between ECM synthesis and degradation [48]. Aggrecan is the primary proteoglycan in articular cartilage, and its loss indicates early OA development [49]. In the present study, abnormal occlusion firstly upregulated Aggrecan and COLII expression levels, and then downregulated them in the cartilage layer of the OD group. When rat MCCs were cultured *in vitro* under hypoxic conditions, the levels of matrix synthesis proteins showed an initial increase followed by a decrease. Knocking out HIF‑1α in chondrocytes led to a significant reduction in Aggrecan and COLII expression under hypoxia, highlighting its critical role in regulating ECM synthesis induced by low oxygen [18].

OA-related cartilage breakdown stems from both ECM degeneration and chondrocyte death. Chondrocytes, the resident cells embedded within the ECM, have a limited capacity for self-repair [50]. This is largely due to the avascular and aneural nature of the cartilage surface, which severely restricts their regenerative potential [51]. Based on the finding that HIF-1α-deficient mice exhibited extensive death of hypoxic chondrocytes within the cartilage, HIF-1α is critical for both sustaining chondrocyte viability under low oxygen and modulating their proliferation [29]. Concordantly, the present study demonstrated that chondrocyte proliferation exhibited a parallel early increase and late decrease *in vivo* and *in vitro*, which is consistent with the expression pattern of HIF-1α. Studies have shown that HIF-1α not only stimulates the anabolism of the chondrocyte ECM by activating hypoxia-induced target genes such as COLII and SOX9 [52,53], but also participates in a series of pathophysiological processes such as cartilage destruction, synovial inflammation, and angiogenesis in osteoarthritis through maintaining anaerobic glycolysis [54].

## Conclusion

The present study findings on the induction of TMJOA through occlusal interference and the *in vitro* hypoxic culture of MCCs demonstrated the expression trend of HIF-1α aligned with that of cell proliferation and ECM formation. Although the genes regulated by HIF-1α in OA were not analyzed, it may be considered that HIF-1α may sustain cellular activity in response to cartilage degradation by promoting chondrocyte proliferation and enhancing ECM production. Overall, the present study demonstrated that the expression of HIF-1α was negatively associated with the damage in an occlusal interference-induced TMJOA rat model. HIF-1α was associated with changes in cell proliferation and ECM production. These findings, by revealing the close relationship between HIF-1α and chondrocyte proliferation and extracellular matrix formation, further deepen our understanding of the pathogenesis of TMJOA and provide potential molecular targets for future disease‑modifying therapies. Several limitations should be acknowledged. First, the absence of functional manipulation of HIF-1α (e.g., via siRNA knockdown, pharmacological inhibition, or overexpression) and the lack of gain- or loss-of-function experiments, which should be addressed in future research to validate the mechanistic role of HIF-1α. Second, the interpretation of the quantitative data in the present study was constrained by the limited sample size (n = 3 per group), which reduced statistical power.

## Supporting information

S1 File Original uncropped and unadjusted images underlying all blot. (ZIP)

S1 Fig The expression of HIF-1α after hypoxic culture of MCCs. The protein and semi-quantitative statistical diagram of HIF-1α in hypoxic chamber, ns = not statistically significant, *P < 0.05, ** *P* < 0.01, n = 3.
(TIF)
